# Neurogenic Aging After Spinal Cord Injury: Highlighting the Unique Characteristics of Aging After Spinal Cord Injury

**DOI:** 10.3390/jcm13237197

**Published:** 2024-11-27

**Authors:** Brittany L. Tretter, David R. Dolbow, Vincent Ooi, Gary J. Farkas, Joshua M. Miller, Jakob N. Deitrich, Ashraf S. Gorgey

**Affiliations:** 1 College of Osteopathic Medicine, William Carey University, Hattiesburg, MS 39401, USA; btretter528163@student.wmcarey.edu (B.L.T.); ddolbow@wmcarey.edu (D.R.D.); vooi585525@student.wmcarey.edu (V.O.); 2 Physical Therapy Program, William Carey University, Hattiesburg, MS 39401, USA; 3 Department of Physical Medicine and Rehabilitation, Miller School of Medicine, University of Miami, Miami, FL 33136, USA; gjf50@med.miami.edu; 4 The Miami Project to Cure Paralysis, Miller School of Medicine, University of Miami, Miami, FL 33136, USA; 5 Christine E. Lynn Rehabilitation Center for the Miami Project to Cure Paralysis, Miami, FL 33136, USA; 6 Department of Kinesiology and Nutrition, College of Applied Health Sciences, University of Illinois Chicago, Chicago, IL 60612, USA; joshuam@uic.edu; 7 Spinal Cord Injury and Disorders Center, Richmond VA Medical Center, Spinal Cord Injury & Disorders Service, 1201 Broad Rock Blvd, Richmond, VA 23249, USA; jakob.deitrich@va.gov; 8 Department of Physical Medicine and Rehabilitation, School of Medicine, Virginia Commonwealth University, Richmond, VA 23298, USA

**Keywords:** neurogenic accelerated aging, neurogenic obesity, neurogenic osteoporosis, accelerated aging

## Abstract

Emanating from several decades of study into the effects of the aging process after spinal cord injury (SCI), “accelerated aging” has become a common expression as the SCI accelerates the onset of age-related pathologies. However, the aging process follows a distinct trajectory, characterized by unique patterns of decline that differ from those observed in the general population without SCI. Aging brings significant changes to muscles, bones, and hormones, impacting overall physical function. Muscle mass and strength begin to decrease with a reduction in muscle fibers and impaired repair mechanisms. Bones become susceptible to fractures as bone density decreases. Hormonal changes combined with decreased physical activity accelerate the reduction of muscle mass and increase in body fat. Muscle atrophy and skeletal muscle fiber type transformation occur rapidly and in a unique pattern after SCI. Bone loss develops more rapidly and results in an increased risk of fractures in body regions unique to individuals with SCI. Other factors, such as excessive adiposity, decreased testosterone and human growth hormone, and increased systemic inflammation, contribute to a higher risk of neuropathically driven obesity, dyslipidemia, glucose intolerance, insulin resistance, and increasing cardiovascular disease risk. Cardiorespiratory changes after SCI result in lower exercise heart rates, decreased oxygenation, and mitochondrial dysfunction. While it is important to acknowledge the accelerated aging processes after SCI, it is essential to recognize the distinct differences in the aging process between individuals without physical disabilities and those with SCI. These differences, influenced by neuropathology, indicate that it may be more accurate to describe the aging process in individuals with chronic SCI as neurogenic accelerated aging (NAA). Research should continue to address conditions associated with NAA and how to ameliorate the accelerated rate of premature age-related conditions. This review focuses on the NAA processes and the differences between them and the aging process in those without SCI. Recommendations are provided to help slow the development of premature aging conditions.

## 1. Introduction

Annually, spinal cord injuries (SCI) affect between 250,000 and 500,000 individuals worldwide [1], with approximately 18,000 new traumatic SCIs reported in the United States alone [2]. SCI wholly or partially disrupts the transmission of neural signals below the injury, resulting in disability that significantly impacts an individual’s independence [1]. Due to medical advances, people with SCI live longer than a century ago with lifelong chronic disability [3]. SCI-related changes result in a functional decline that accelerates aging in this population, serving as a neurogenic model for premature aging [4,5]. Such premature aging is attributed to additional stresses, surpassing the body’s self-repair capacity [5].

Physiological function generally peaks during early adulthood despite variations in individual aging processes. Subsequently, a gradual decline occurs, characterized by a significant reduction in organ system reserve capacity. As these reserves diminish to less than 40% of original functioning, individuals become increasingly susceptible to injury, infection, and disease [5,6]. Individuals with SCI are at a higher risk of developing age-related conditions, resulting in the conclusion that individuals with significant chronic SCI experience an accelerated or premature aging process compared to those without disabilities [5,6,7,8,9]. Hitzig and associates [5] completed an evidence-based review of aging on numerous body systems following SCI and found that the musculoskeletal, cardiovascular, and endocrine systems significantly undergo premature aging. While it is important to recognize the accelerated aging process after SCI, it is essential to recognize the distinct differences in the aging process between individuals without physical disabilities and those with SCI. These differences, influenced by neuropathology, indicate that it may be more accurate to describe the aging process in individuals with chronic SCI as neurogenic accelerated aging (NAA).

This review highlights the differences between the aging processes of those with SCI and those without SCI. Understanding the long-term effects of NAA on individuals with SCI is crucial for systematically evaluating the process and developing interventions to improve outcomes and enhance the quality of life for those living with SCI.

## 2. Body Composition Changes

### 2.1. Skeletal Muscle

The general aging process gives rise to cellular and molecular changes that lead to decreased muscle size and the capacity to generate force [10]. Moreover, alterations within myofibers lead to deficiencies across diverse physiological realms, encompassing muscle fiber activation, excitation–contraction coupling, interactions between actin and myosin cross-bridges, energy generation, and repair and regeneration processes [10]. SCI results in upper and lower motor neuron injuries, resulting in a full or partial interruption of nerve conduction within the central and/or peripheral nervous systems. Upper motor neuron injuries create loss of functional use and muscle wasting, often accompanied by spasticity, which can create further dysfunction [11]. Lower motor neuron injuries produce hypotonicity along with muscle wasting due to injury to the peripheral motor neurons [12]. While muscle atrophy is a natural occurrence over time with aging, skeletal muscle loss after SCI is faster and greater due to the combination of skeletal muscle denervation, immobilization due to paralysis, and disuse due to decreased physical activity levels [11,13]. As individuals without SCI age, there is a gradual decline in muscle mass, particularly in the lower limbs, accompanied by a 30% to 40% reduction in muscle fiber from the second to the eighth decades of life [10]. The decrease in muscle fiber size varies by fiber type, with a noticeable 10–40% reduction in the size of type II fast twitch fibers observed in muscle tissue from elderly individuals compared to younger controls [10]. Conversely, after SCI, there are rapid increases in muscle atrophy and major changes in muscle fiber type [8]. Aging individuals without SCI lose predominantly type II fast twitch glycolytic fibers, while type I slow twitch oxidative fibers remain relatively unchanged [10]. These specific alterations in fiber types in the general population can be attributed to the age-related restructuring of motor units, predominantly leading to the denervation of type II muscle fibers and the subsequent re-innervation of type I slow twitch muscle fibers [10]. However, fiber-type transformation is the opposite for individuals with SCI. Several studies have examined muscle composition in individuals with SCI, ranging from 10 months to 10 years post-injury. These studies have identified that the paralyzed muscles of the vastus lateralis, gastrocnemius, and soleus were predominantly composed of type II fast-twitch glycolytic muscle fibers [14,15]. Similarly, Crameri et al. [16] completed a case series involving two individuals with SCI and found that after one month, the untrained paralyzed skeletal muscle exhibited a notable decline in type I fibers from 50% initially to 9%. The myosin heavy chain I (slow twitch) decreased from 27% to 6%. The cross-sectional area (CSA) of all fiber types was diminished. However, the predominant fibers remaining were type IIX fast-twitch glycolytic muscle fibers, the inverse of what was found in older individuals without SCI. An early study by Scelsi et al. [17] used muscle biopsies up to 4 months post-SCI and found no change in the relative percentage of type I and II fibers, whereas 7 and 9 months after the injury, there was a notable decrease in type I fibers. Furthermore, results found by Lotta et al. [18] in the early stages of paraplegia (1–6 months) displayed pronounced atrophy primarily in type IIA fibers in the medial head of the gastrocnemius and soleus muscles. However, as paraplegia progressed into the long term (8–10 months), a decline in type I fibers was noted, accompanied by further atrophy. Both muscles exhibited a significant presence of type IIX fibers, suggesting a shift towards fast-twitch glycolytic fiber types. There was also a consistent occurrence of IIX intermediate fibers in both muscles throughout the extended duration of paraplegia. This may lead to a reduction in mitochondrial density and activity, as demonstrated by a decrease in citrate synthase and, subsequently, mitochondrial dysfunction. Mitochondrial dysfunction after SCI is prominent after the age of 40 years and typically associated with increasing ectopic adiposity and dysregulation in metabolic profile [19,20]

In the aftermath of SCI, muscles affected by upper motor neuron paralysis experience a marked alteration from the usual mixture of slow twitch type I and fast twitch type II fibers to predominantly fast twitch glycolytic type II fibers. Burnham and associates [21] suggested a shift in muscle characteristics that may begin within 4 to 6 weeks following the SCI with a time constant of 4.7 months, and approximately 62% of the fibers transformed during this period. Moreover, as type I fibers transition to type II, they enter a transitional phase, simultaneously expressing both slow and fast myosin heavy chain isoforms. Enzymatic activity associated with oxidative metabolism declines within the first few months post-SCI, potentially contributing to the shift towards faster muscle fibers. By 70 months, the fiber types displayed are almost entirely type II fast-twitch glycolytic fibers. Consequently, paralyzed muscles’ overall strength and endurance diminish significantly [14].

**Figure 1:**
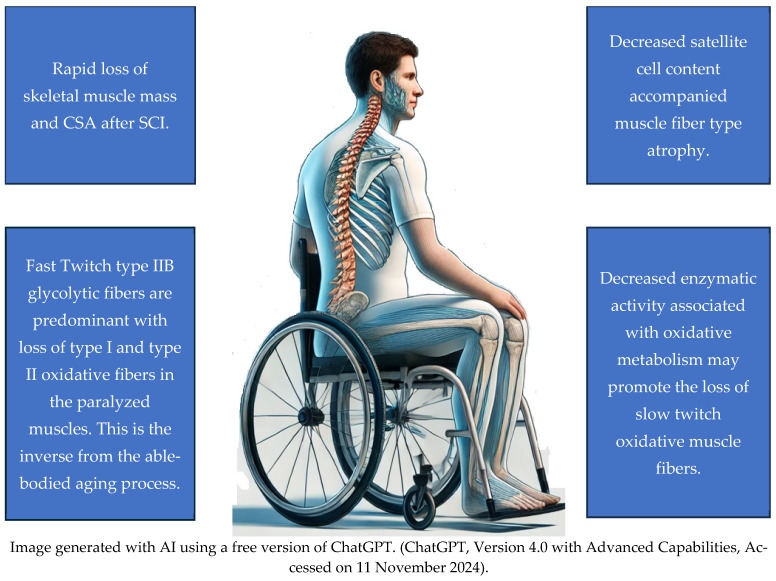
Skeletal Muscles Changes after SCI.

Despite similar manifestations of muscle atrophy, strength loss, and decreased function in both aging individuals without SCI and those with SCI, the underlying physiological processes differ in terms of the speed of atrophy, muscle fiber size, and the content of satellite cells. Verdijk et al. [22] used muscle biopsies to investigate cellular differences between eight young men with SCI, eight young men without SCI, and eight healthy elderly adults. The results elucidated the pivotal role of satellite cells as the progenitors of various myofiber types. The reduction in satellite cell count was consistent with the atrophy of the corresponding fiber types, highlighting the intricate relationship between satellite cells and myofiber types. Notably, the study encountered a scarcity of type I muscle fibers in SCI biopsies, which limited the analysis to only three of the eight SCI participants. There was a marked decrease in the percentage of type I muscle fibers and the area percentage occupied by type I muscle fibers in individuals with SCI compared to both young and elderly subjects without SCI. Moreover, there was a reduction in type II muscle fiber size in non-SCI older men, indicative of specific type II muscle fiber atrophy with natural aging. Individuals with SCI significantly decreased muscle fiber size for both type I and II fibers compared to young controls, with atrophy exceeding 50%. Additionally, muscle fiber type composition shifted towards approximately 90% of the type II muscle fibers in SCI subjects, contrasting with the lack of such a shift in the non-SCI elderly participants. Furthermore, the study confirmed and extended previous findings on the decline in satellite cell content accompanying type II muscle fiber atrophy with aging. This decline was similarly observed in subjects with SCI, underscoring the detrimental impact of severe physical inactivity on muscle health and highlighting the importance of targeted interventions in mitigating muscle atrophy in these populations. Satellite cells are important skeletal muscle stem cells associated with the maintenance of skeletal muscle [23]. It is interesting to note that when neuromuscular electrical stimulation resistance training (NMES-RT) was introduced with testosterone treatment, there was a paradigm shift in the contractile properties of the muscle from fast-fatigable to more fatigue resistance as characterized by increasing muscle quality, fiber CSA, increase in mitochondrial citrate synthase and succinate dehydrogenase, slowness of the rise time, and finally the number of myonuclei [24,25,26]. These findings highlight the critical importance of implementing targeted rehabilitation exercises to address the accelerated muscle atrophy and functional decline observed in individuals with SCI. Understanding the specific muscle deterioration processes after SCI can help develop strategies to better mitigate the effects of rapid muscle degeneration and enhance functionality in this population (Figure 1).

#### Recommendations for Maintaining Skeletal Muscle

Increased physical activity is recommended to slow the NAA effects on skeletal muscle after SCI. Resistance training has been well established to maintain muscle mass during aging. Regarding paralyzed muscles, functional electrical stimulation exercises, including neuromuscular electrical stimulation resistance training, functional electrical stimulation cycling, and rowing, have been shown to maintain or increase muscle mass after SCI, with neuromuscular electrical stimulation resistance training being the most effective when applied to specific muscle groups [27,28]. Exercise has also been shown to increase the number of satellite cells in muscles, with greater exercise intensities and volumes being key to decreasing the rate of satellite cell loss with aging [29]. Because there is a shift away from oxidative muscle fiber types after SCI, adding aerobic-type activities to an exercise program would be advisable.

### 2.2. Bone Health

The patterns of bone loss and fracture susceptibility differ significantly between individuals with SCI and those without SCI, highlighting unique challenges in bone health management (Table 1). As individuals without SCI age, their risk of fractures escalates, with reports suggesting a tenfold increase in fracture risk over a decade [29]. The vertebrae, proximal femur, distal forearm, and shoulder are common sites for osteoporosis diagnosis and increased risk of fracture in persons without paralysis [30]. Many individuals without SCI initially report height loss due to vertebral compression from fractures, which can be objectively assessed by increasing occiput-to-wall distance due to dorsal kyphosis [30]. Bone mineral density (BMD) diminishes with age in both genders and is commonly viewed as an indicator of fracture susceptibility [29]. Studies reveal that cortical bone becomes increasingly brittle and weaker as individuals without SCI age [29]. Microscopic damage accumulates with age and is characterized by escalating micro-crack densities and lengths [29]. Within trabecular bone, the diminishing trabecular surface amplifies stress and bone deformation, escalating fracture risk, especially in fragile bone [29]. While not uniformly affecting all bones, age-related changes diminish bone deposition per the remodeling cycle, potentially stemming from reduced osteoblast precursor cells, stem cell numbers, or osteoblast lifespan [29]. Osteoblast precursor differentiation and marrow stromal cell proliferation decline with age [29]. Osteocyte apoptosis rises with tissue age, further weakening bone [29].

Bone loss after SCI proceeds in an accelerated manner but has its own unique pattern dissimilar to typical bone loss patterns [31,32]. The rate of bone loss after SCI is associated with the injury duration and the level of injury as opposed to age or gender as in the population without SCI [32]. Neurogenic osteoporosis is a medical condition that rapidly occurs in persons with SCI due to bone resorption caused by mechanical disuse resulting from chronic immobilization and paralysis [31,32,33]. Non-mechanical factors also play a significant role in the development of neurogenic osteoporosis, including hypothalamic disturbances, hypercortisolism, gonadal dysfunction, and other endocrine disorders, insufficient nutritional support, and dysregulated vasoregulation [31,32,33]. The lack of mechanical strain on bone following SCI contributes significantly to bone loss [34]. Due to decreased physical activity and subsequent lack of mechanical stimuli acting on the skeleton system due to paralysis, osteoclastic activity escalates without a proportional rise in osteoblastic activity, inflating the neurogenic osteoporosis process in individuals with SCI [35].

In individuals with SCI, cortical bone not only loses density but also experiences significant geometric deterioration. Modlesky et al. reported a 24% reduction in cortical bone volume and a 53% increase in medullary cavity volume in the SCI group [36]. The study found that cortical walls were 27–47% thinner, while the medullary cavity was over 50% larger and more than 20% wider compared to controls. Additionally, the anterior–posterior width was greater than the medial–lateral width in both groups [36].

In the acute setting after SCI, osteoblastic activity is suppressed as bone resorption increases, accompanied by hypercalcemia, hypercalciuria, and increased alkaline phosphate and osteocalcin, both bone resorption markers [32,35,37]. This cascade of events has been reported to reach a steady state anytime between one and several years post-injury [35,38,39,40]. Individuals with SCI experience calcium depletion at a rate 2 to 4 times higher than those without SCI who endure extended periods of bed rest [35].

Women without SCI show initial bone loss in the lumbar spine, total hip, and femoral neck starting around age 40–44, and men without SCI experience this bone loss at an earlier age, typically between 25–39 [41]. Immediately after an SCI, a rapid decline in BMD occurs within a few days to weeks, approximately 2 to 4 percent per month [42]. A study involving male adults under 40 years of age who experienced SCI found that approximately one-third of bone mass was lost in the lower extremities within the first three to four months and a 40% decline in the first six months following the injury [32,35,39,43]. Individuals with SCI continue to rapidly lose trabecular bone density for 1 to 3 years post-injury and continue to lose cortical bone for the next 10 years [14,35]. It should be noted that trabecular bone encompasses a larger surface area, which leads to a faster turnover rate than cortical bone. Modlesky and colleagues [44] observed elevated trabecular bone loss in the distal and proximal tibia among men with chronic SCI. They concluded that the decreased trabecular bone in the knee leads to increased fracture occurrence after SCI. Slade et al. [45] study with individuals with SCI aged 22–62, the trabecular bone around the knee deteriorates significantly compared to ambulatory women, with unloading having a more profound impact than menopause-related estrogen loss [45]. Another study on trabecular bone concluded that the bone is replaced with fatty marrow, leading to a depletion in trabecular bone [35,46]. On the other hand, cortical bone, which constitutes a significant segment of the long bone shafts, experiences a gradual thinning, leading to a progressive decline in bone strength [35].

Highlighting further differences between individuals without SCI and those with SCI, common fracture sites for individuals without SCI are the lumbar vertebrae and hip, while those with SCI are prone to osteoporosis and increased fracture incidences at the distal and proximal epiphyses of the femur and tibia, with trabecular bone loss estimated to be 52% and 70%, respectively, as compared to those without SCI [35,47]. The most common fracture sites after SCI are the distal femur 35.6% and proximal tibia 20.5% [48]. Post-SCI, 14% of individuals experience fractures within five years of the injury, with the incidence increasing to 28% within ten years and 39% within fifteen years post-injury [35,49]. As time progresses post-injury, individuals become increasingly susceptible to fractures of the lower extremity. Additionally, for individuals with SCI, fractures are more frequently encountered during activities of daily living [35]. The reduction in BMD post-SCI heightens the risk of fractures even with activities like transferring from a chair to a bed [33]. Despite significant loss of BMD observed in persons with SCI, their lumbar vertebrae displayed preservation of BMD, as documented in prior studies [35,50,51]. This is thought to be due to weight loading on the vertebrae through sitting as compared to loss of weight bearing on the legs [39,51].

In addition to BMD levels, other important factors such as bone micro-architecture, bone geometry, and bone marrow adiposity should be taken into consideration as a holistic review for the prognosis and prevention of fractures. SCI patients will have significant geometric changes in their bone with altered CSA and a reduction of cortical thickening. These changes can greatly reduce the overall mechanical strength of their bones, increasing the risk of injury [36]. SCI patients may also experience fracture despite having a normal BMD due to the loss of trabecular connectivity and structure, altering the bone micro-architecture [45]. The severity of trabecular bone deterioration is even greater in SCI patients compared to the high-risk populations of postmenopausal women [45]. Furthermore, findings within Gorgey et al. [52] reinforced the idea that bone marrow adiposity is an important factor that contributes to bone fragility in SCI patients. The investigation depicted that men with motor-complete SCI had increased deposition of adipose tissue within femoral bone marrow cavities. This accumulation of fat and increased yellow bone marrow adiposity CSA can interfere with bone formation and lower cortical bone CSA [52]. Hence, bone health should not be assessed solely by BMD, but it is equally important to understand the conditions of bone micro-architecture, bone geometry, and bone marrow adiposity in order to develop comprehensive treatments in the SCI population.

#### Recommendations for Maintaining Bone

Recommendations to slow the NAA of bone after SCI include weight-bearing activities that work best during the acute stages to slow bone loss. High-intensity electrical stimulation activities that take several months to a year or more can provide minor to moderate improvements in bone restoration. Bone restoration is a slow process, and thus far, only minimal benefits have been displayed through physical activities alone [53,54]. Non-exercise-related strategies include supplementation of vitamin D, which has been shown to be beneficial for individuals who also have a deficiency in vitamin D but not for those with SCI without the deficiency, and the use of bisphosphonates. Medications that fall under the bisphosphonate name include Etidronate, Clodronate, Pamidronate, Tiludronate, and Alendronate [35]. These medications have been shown to be successful in reducing hip fractures but not fractures above and below the knee [35]. Because there are limitations to all the treatment strategies for decreasing bone loss and the risk of fractures after SCI, a combination of pharmacological, vitamin, and exercise treatments is often recommended.

### 2.3. Adipose Tissue

As individuals without SCI age, there is a noticeable decline in basal and resting metabolic rate, ranging from 5% to 25%, contributing significantly to weight gain and increased body fat accumulation [55]. The gradual increase in body fat typically begins around 20 to 25 years of age and persists until around 65 years [55]. Furthermore, fat redistribution is particularly towards the abdominal region, with infiltration into muscle and bone [55]. This phenomenon underscores the complex interplay between metabolic changes and the structural alterations occurring within various body compartments over time [55]. Neurogenic obesity includes many changes after SCI that detrimentally alter energy balance, creating a harmful lean mass-to-fat mass ratio (LM to FM) (Table 2) [56]. These alterations include a reduction in LM, anabolic deficiency, sympathetic dysfunction, and blunted satiety [56]. Individuals with complete SCI experience a profound reduction in LM and an increase in FM compared to individuals without SCI [57]. Spungen et al. [57] investigated body composition changes in individuals with SCI and demonstrated a 13% higher FM per unit of body mass index (BMI) than their matched non-SCI counterparts. Central adipose tissue can be further classified into subcutaneous adipose tissue (SAT) and visceral adipose tissue (VAT) [58]. VAT, including liver adiposity, has been linked with an increased propensity to develop insulin resistance, dyslipidemia, and other metabolic disorders [59,60]. Visceral adipose tissue (VAT) plays a crucial role in releasing proinflammatory adipokines such as tumor necrosis factor-α (TNF-α), interleukin-6 (IL-6), plasminogen activator inhibitor-1 (PAI-1), and thrombin activatable fibrinolysis inhibitor-1 (TAFI-1) [61]. These substances are typically elevated in individuals with obesity, both in individuals without SCI and in those with SCI, contributing to systemic inflammation. Persons with SCI have increased levels of circulating inflammatory cytokines, as demonstrated by significantly higher levels of IL-6 in obese individuals with SCI than individuals without SCI, with a positive correlation observed between IL-6 and fasting insulin, particularly in those with SCI. Additionally, plasma levels of PAI-1 positively correlate with abdominal obesity in chronic SCI. In contrast, abdominal sagittal diameter is associated with high sensitivity C-reactive protein (hs-CRP), a marker of systemic inflammation stimulated by IL-6 and TNF-α and found to be elevated in chronic SCI. In a monozygotic twin model study, SCI-afflicted twins exhibited a 7% increase in FM per unit of BMI and a 10 kg decrease in LM in the lower limbs relative to their healthy counterparts [62]. Over five years, twins with SCI experienced a marked decline in fat-free mass (FFM), averaging nearly 4 kg, while regions above the injury level remained unchanged. Intriguingly, twins with acquired paraplegia exhibited significantly higher total body FM and fat percentage per unit BMI compared to their non-SCI counterparts. Among the findings, the twins with SCI displayed a striking contrast, harboring 5 kg more whole-body fat mass and 7% higher whole-body fat percentage compared to their non-SCI counterparts, regardless of the unit of BMI.

SCI induces a catabolic state, gradually depleting total body lean tissue [62]. Their findings suggest that this loss in lean body tissue predominantly occurs in the legs of individuals with lower SCI, with a staggering rate of approximately 0.8 kg per decade, nearly double the pace observed in matched controls [62]. According to Gorgey and Dudley, intramuscular fat (IMF) in the thigh muscle groups increased by 126% within three months following a six-week period after an incomplete spinal cord injury [63]. Monroe et al. [64] demonstrated a contrast in FFM between those with SCI and the individuals without SCI, with those with SCI exhibiting lower FFM levels compared to the controls (69.2 ± 8.7 kg versus 77.2 ± 7.2 kg). Participants with SCI displayed higher FM (30.8 ± 8.7 kg versus 22.8 ± 7.2 kg) despite having a similar BMI to individuals without SCI. Gater et al. [65] demonstrated the epidemic nature of neurogenic obesity in the SCI population by completing a cohort body composition study on 72 individuals with chronic SCI using the criterion standard four-compartment model. They reported neurogenic obesity in 97% of the cohort. McMillian and colleagues [66] described the complex processes involved in the development of neurogenic obesity after SCI reported how sensory nerves communicate with adipose tissues to play a role in informing the central nervous system and regulating the autonomic nervous system, which is closely linked to the functioning of the hypothalamus. After SCI, denervation of these sensory fibers in adipose tissues may inhibit these processes, disrupting metabolic balance.

#### Recommendations for Lowering Body Fat

Recommendations to decrease the effects of NAA on adiposity should be multifaceted. Due to the link between adiposity and all-cause mortality, obesity is a legitimate therapeutic target. Based on feasibility and associated risk, a diet and exercise therapy trial is recommended. Optimally, the combination of diet and exercise needs to induce a negative energy balance; however, because SCI reduces muscle mass and oxidative capacity, this can be difficult without the use of electrical stimulation activities that engage the paralyzed muscles of the legs [67]. Hybrid activities such as voluntary arm cycling combined with functional electrical stimulation cycling could provide larger muscle mass activation for physical activity, although many may not have access to this type of equipment. Because of the associated health issues, morbid obesity may not respond fully to diet and exercise alone; thus, pharmacological or bariatric surgery followed by diet and exercise may be a viable option [68].

## 3. Endometabolic

In the natural aging process of individuals without SCI, the development of metabolic syndrome is predominantly ascribed to the aging process itself [69]. With advancing age, individuals tend to experience increased insulin resistance, a critical component of metabolic syndrome [69]. This insulin resistance manifests in unregulated gluconeogenesis, heightened adipose lipogenesis, and impaired glucose uptake in skeletal muscles [69]. Research has consistently shown a heightened occurrence of metabolic syndrome in individuals with SCI [70] (Table 3).

From the third to the seventh decade of life for individuals without SCI, there is a notable increase in VAT deposition [69]. An increase in intra-abdominal fat predisposes individuals to impaired insulin action in the liver, which can ultimately lead to the development of type 2 diabetes. A study involving 22 men with complete paraplegia found that larger abdominal sagittal diameters were associated with adverse metabolic markers such as elevated fasting glucose, increased fasting and post-load insulin levels, higher estimates of insulin resistance derived from the Homeostasis Model Assessment, lower levels of high-density lipoprotein cholesterol (HDL-C), elevated triglycerides, and increased CRP levels [65]. The Paralyzed Veterans of America conducted a nationwide survey of veterans and found a 20% prevalence of diabetes among individuals with SCI, a rate three times higher than that of the general population [71]. Glucose tolerance is also reported to be lower post-SCI, which suggests an early onset of diabetes mellitus in individuals with SCI compared to their counterparts without SCI. The elevated intramuscular fat content observed in SCI patients has been linked to contribute to their impaired glucose intolerance [72].

Between the ages of 30 and 45 years for individuals without SCI, declines in resting energy expenditure (REE) and alterations in body composition began to manifest [73]. These changes included decreases in REE adjusted for skeletal muscle and organ mass, as well as adipose tissue, by approximately 145 kJ/day per decade in women and 604.8 kJ/day per decade in men, following the respective ages of 35.2 and 34.3 years [73]. Individuals with SCI exhibit a lower measured metabolic rate compared to those without disabilities. The average metabolic rate among individuals with long-term SCI typically falls within the range of 1256 to 1854 kcal/day, which shows minimal overlap with the metabolic rate reported for individuals without disabilities, ranging from 1594 to 2249 kcal/day [73]. Bauman and associates [74] studied thirteen pairs of monozygotic twins discordant for SCI, which further highlighted metabolic differences, showing that the measured basal energy expenditure or resting energy expenditure was significantly lower in the twins affected by SCI compared to their non-SCI counterparts.

Independent associations between low testosterone levels and non-alcoholic fatty liver disease (NAFLD) have been identified in individuals with SCI, with each decrement in testosterone associated with an increased risk of NAFLD [75]. Patients diagnosed with NAFLD also exhibited higher triglyceride concentrations and insulin resistance, aligning with metabolic syndrome components observed in individuals without SCI with NAFLD [75]. Testosterone and human growth hormone (HGH) secretion have been shown to be diminished in individuals with SCI compared to those without SCI [76,77].

As an individual ages, there is a significant reduction in HGH secretion, leading to a corresponding decrease in insulin-like growth factor 1 (IGF-1) concentrations [64]. Reduced peripheral levels of IGF-1 in humans have been linked to higher risks of various conditions, such as type 2 diabetes mellitus and cardiovascular disease [76,77,78,79]. Rincon et al. [76] noted that young individuals with SCI had a reduction of the HGH/IGF-I axis, which impedes the capacity for cellular repair and leads to a state of premature aging [76,78,79]. The implications of a relative HGH deficiency might exacerbate the adverse alterations in body composition associated with SCI-induced paralysis and immobility [76,78,79]. Table 4 highlights the major endometabolic changes associated with aging after SCI.

### Recommendations for Decreasing Cardiometabolic Risk

Similar to addressing excessive adiposity after SCI, addressing the various factors contributing to the risk of metabolic diseases such as diabetes, dyslipidemia, systemic inflammation, and other conditions affected by aging, it is important to make healthy lifestyle changes. These changes should include dietary adjustments and regular physical activity. Similar to reducing body fat, exercise must provide enough muscle activity to create sufficient stress on the cardiovascular system and improve central and peripheral vascular health. Hybrid-type activities that involve the arms voluntarily and the legs through electrically evoked exercise are a possible option. If additional measures are required beyond lifestyle changes involving regular exercise and a healthy diet, pharmacotherapy and bariatric surgery can be considered as options, but they also come with unique risks and varying benefits for individuals with SCI [80].

## 4. Cardiovascular

In the general non-SCI population, the cardiovascular system undergoes gradual deleterious changes as we age [81,82]. Many factors, including genetics, the environment, and lifestyle choices, may affect cardiovascular health; however, aging has been reported as the most dominant risk factor for developing cardiovascular disease [82]. The aging process typically involves stiffening of blood vessels, which leads to atherosclerosis, the building up of plaque in the vessels that may result in blockages and increased vascular inflammation that is also associated with atherosclerosis and the increased risk of heart disease [81,82]. However, SCI produces a number of additional factors that significantly degrade cardiovascular health and create a significant disparity in cardiovascular health between individuals with SCIs and those without SCIs. Obesity, low levels of physical activity, increased systemic inflammation, dyslipidemia, reduced venous blood return to the heart due to the lack of muscle contractions in the legs, autonomic dysfunction, and hypotensive responses to activity are associated with decreased cardiovascular function and increased risk of cardiovascular disease [5,8,9,66,76,83]. Table 5 highlights the major changes in the cardiovascular system as a result of NAA in persons with SCI. Lavis et al. [83] described a physiological disparity between individuals without SCI and those with SCI in the circulation of blood during physical activity. The population without SCI experiences a venous pumping action of the muscles in the lower extremities and sympathetic-mediated input that maintains systemic blood pressure and flow. As a result, there is an increase in the cardiac end-diastolic volume, which increases the heart’s stroke volume (SV) and cardiac output (CO). This effect enables a more significant delivery of oxygenated blood to the active muscles to compensate for their increased metabolic rate. However, this process is significantly impaired in individuals with SCI. This is due to the loss of lower extremity muscle activation due to paralysis, leading to alterations in blood flow throughout the cardiovascular system. The loss of the veno-muscular pumping action by the muscles of the lower legs, combined with changes in the autonomic nervous system (particularly the sympathetic system), further affects the cardiovascular response during physical activity. Those with higher-level thoracic and cervical injuries may also experience a partial or complete separation of the autonomic nervous system from central control, which can impair peak cardiac output [83].

The loss of venous pumping and compensatory vasoconstriction can lead to blood pooling in the lower extremities, resulting in decreased SV at rest and during exercise. This, in turn, impairs CO [83].

Furthermore, elevated plasma homocysteine levels, particularly pronounced in older adults with SCI, are associated with increased vascular disease risk, and heightened CRP levels in individuals with SCI suggest a potential link to altered lipid profiles and increased atherogenesis risk [79]. Individuals with SCI exhibit a more significant atherosclerotic burden compared to their non-SCI counterparts. Adults with SCI have a significantly higher incidence of cardiometabolic morbidities compared to those without SCI, with survival analyses revealing a greater hazard for various cardiometabolic disorders, including NFALD and heart failure [79]. These findings underscore the critical need for targeted interventions to mitigate cardiovascular risk and improve outcomes in individuals living with SCI.

Peterson et al. [79] reported that altered autonomic control in individuals with SCI is demonstrated by abnormal heart rate responses during exercise in paraplegic individuals and increased mean arterial blood pressure in those with tetraplegia. These physiological changes contribute to cardiovascular health modifications in SCI populations.

Individuals with SCI face an increased risk of developing coronary heart disease at a younger age compared to the general population [84]. Heart disease has become a leading cause of mortality among those with long-term SCI, accounting for more than 20% of deaths and approximately 46% of deaths in individuals who live with SCI long-term (≥30 years) [83,84]. Risk factors associated with cardiovascular disease in the aging SCI population are metabolic alterations characterized by low levels of HDL-c, glucose intolerance, insulin resistance, and reduced metabolic rate [5,8,9,65,79,82]. Reduced HDL-c levels are particularly prevalent, and individuals with complete tetraplegia exhibit the lowest values and highest risk for cardiovascular issues [84]. Individuals with SCI show a greater incidence of atherosclerosis compared to individuals without SCI [5]. Bauman and colleagues [85] conducted a study on 100 veterans with SCI, evaluating their lipoprotein profile. They found that 37% of participants had an HDL-c lower than 35 mg/dL, 18% had low-density lipoprotein cholesterol (LDL-c) greater than 160 mg/dL, and that dyslipidemia increases an individual’s risk factor for coronary artery disease. Premature changes in body composition, such as sarcopenia and increased adiposity, are associated with cardiovascular deconditioning [61,86]. Inflammation markers like IL-6 and CRP are related to heart disease and type 2 diabetes [61,86]. Even slight elevations in IL-6 and CRP levels, which may not manifest clinically, can heighten the risk of developing heart disease and type 2 diabetes in individuals who were previously considered healthy [61,86]. A study involving 22 men with complete paraplegia (mean age, 39 years) exhibited median IL-6 levels 42% higher and median CRP levels 62% higher compared to healthy men (mean age 59 years) without disabilities who remained free of chronic diseases over a 6-year follow-up period [61].

Individuals with tetraplegia often have lower CO and reduced risk of hypertension due to sympathetic dysfunction, while paraplegic individuals, especially those with mid-thoracic cord injuries, may experience hypertension related to inactivity and obesity [84]. Two separate investigations unveiled notable insights into autonomic control among individuals with SCI in contrast to individuals without SCI. Specifically, males with complete T6 or above paraplegia had an atypical (absent) heart rate response during exercise, while men with complete tetraplegia displayed an elevated mean arterial blood pressure [5]. These observations indicate an altered autonomic control, potentially influencing the integrity of the SCI cardiovascular system in those with SCI [5].

### Recommendations for Decreasing Cardiovascular Risk

Recommendations for countering the effects of NAA on the cardiovascular system include aerobic-type activities. Because of the predominance of fast-twitch glycolytic muscle fibers, it is possible that interval training with high-intensity exercise interspersed with a low-intensity or resting phase would be beneficial. Hybrid activities that provide electrically evoked exercise with the paralyzed lower extremities combined with voluntary arm exercise provide more muscle activation, which can lead to more intense exercise and potentially greater aerobic conditioning [87]. Hodgkiss et al. [88] completed a meta-analysis and determined that exercise interventions can significantly improve cardiorespiratory fitness in individuals with SCI by a clinically meaningful greater than one adjusted metabolic equivalent (≥2.7 mL/kg/min), which equates to a reduction in cardiovascular risk. More research needs to be completed to verify the range of benefits of exercise activities. For all exercise programs, participants need to be screened by their physician prior to starting and follow the guidelines provided by specialists in the field. This will increase the benefits and safety [87]. Some interventions that may help improve venous blood return to the heart during activity include lower extremity compression garments, abdominal binders, and lower extremity functional electrical stimulation [83,88,89,90]. Other lifestyle choices beyond regular exercise that may help reduce cardiovascular risk include a heart-healthy diet and pharmacological treatment of cardiovascular risk factors such as hypertension and dyslipidemia.

## 5. Possible Therapies and Anti-Aging Treatments for SCI

Activities combining electrical stimulation and exercise with the extremities have shown promise in potentially reversing the aging effects of SCI on body composition and cardiovascular health [25,54,67,89,90]. Diet also remains a critical factor in determining overall health in SCI individuals. A commitment to an improved diet and lifestyle is required for the long-term success of any intervention for any person with SCI [68].

Stem cell implantation is another approach that has produced promising results in both rodent and human models. In rat SCI models, implantation of induced pluripotent stem cells led to improved locomotor function and prevented further damage to the spinal cord [91]. Fessler et al. have validated the safety of stem cell implantation, identifying no adverse events or further neurological decline in individuals with SCI over a 10-year period after implantation [92]. They also advocated for the safety of 3 escalating doses of oligodendrocyte progenitor cells after identifying no further neurological damage, with some individuals even recovering partial function [93]. Stem cells have the potential to drastically change the landscape of SCI and have produced some encouraging results in various studies, but further research is still required to determine their ability to treat or reverse the effects of SCI.

## 6. Conclusions

The term “accelerated aging” is commonly linked with the early onset of systemic pathologies after SCI. Individuals with SCI experience a pattern of decline during aging that is different from individuals without SCI. Muscle atrophy and skeletal muscle fiber type transformation occur rapidly and in unique patterns after SCI, resulting in decreased skeletal muscle CSA and a predominance of type II fast-twitch glycolytic fibers. Bone loss also develops more rapidly and in an extraordinary bone loss pattern, with the greatest risk of osteoporosis and fracture risk at the distal femur and proximal tibia. Other factors such as excessive adiposity (especially VAT), decreased testosterone and HGH, and increased systemic inflammation contribute to a higher risk of cardiometabolic diseases and neurogenic obesity. Cardiovascular changes after SCI result in lower exercise heart rates and decreased oxygenation in the body, neuropathically driven dyslipidemia, glucose intolerance, insulin resistance, and a reduced metabolic rate, increasing the risk of cardiovascular disease. Research should continue to focus on addressing conditions associated with NAA to ameliorate the accelerated rate of premature age-related conditions.

## Figures and Tables

**Table 1:** Changes in bone health after SCI.

Effects of SCI on Bone
Rapid bone loss pattern unique to SCI with rate of bone loss dependent on time since injury and level of injury, not gender or age.
Increased bone resorption due to mechanical disuse resulting from chronic immobilization and disuse due to paralysis.
Most prominent loss of bone mass and increased risk of fracture in distal and proximal tibia and femur. Vertebral bone is maintained.
Hypothalamic disturbances, hypercortisolism, gonadal dysfunction and other endocrine disorders, insufficient nutritional support, and dysregulated vasoregulation also impact bone loss.

**Table 2:** Adiposity changes after SCI.

Effects of SCI on Adiposity
SCI creates profound reductions in lean mass (LM) and an increase in fat mass (FM) compared to non-disabled individuals (VAT and SAT).
SCI results in a reduction in lean mass (muscle and bone), anabolic deficiency, sympathetic dysfunction, and blunted satiety
Fat cells release proinflammatory adipokines such as TNF-α, IL-6, PAI-1, and TAFI-1.
SCI may disrupt neural signals between adipose tissue and the CNS and SNS, undermining homeostasis.

**Table 3:** Metabolic syndrome rates after SCI.

Metabolic Syndrome (Adapted from Gater et al., 2021) [67]	n (%)
Metabolic syndrome = 3 abnormal measures out of the 5 from the following list.	3/5
SCI-Specific BMI ≥ 22 kg/m^2^ (n = 72)	59 (82%)
4 Compartment Model % body fat	
All (n = 72)	70 (97%)
≥22 for men (n = 59)	59 (100%)
≥35 for women (n = 13)	11 (85%)
Triglycerides ≥ 150 mg/dL or under Treatment (n = 70)	23 (33%)
High-density lipoprotein cholesterol < 40 (men) or <50 (women) mg/dL or under Treatment	
All (n = 72)	60 (83%)
<40 for men (n = 59)	50 (85%)
<50 for women (n = 13)	10 (77%)
Systolic blood pressure ≥ 130 or Diastolic blood pressure ≥ 85 mmHg, or under Treatment (n = 72)	31 (43%)
Fasting Glucose ≥ 100 mg/dL or under Treatment (n = 71)	23 (32%)

**Table 4:** Endometabolic changes with aging after SCI.

Effects of SCI on Metabolism
It is well established that obesity and metabolic syndrome are epidemic in the SCI population.
Increased intra-abdominal fat increases risk of type 2 diabetes, dyslipidemia, and increased systemic inflammation.
Decreased skeletal muscle and reduced physical activity levels decreases in individuals with SCI resulting in increased adiposity.
SCI decreases testosterone and HGH levels which are associated with NAFLD. Those with NAFLD exhibit increased triglyceride s and insulin resistance.

**Table 5:** Cardiovascular changes with aging after SCI.

Effects of SCI on Cardiovascular Health
Obesity and low physical activity levels after SCI promote increased risk of cardiovascular disease at greater levels and younger ages that in the able-bodied population
Increased plasma homocysteine, CRP and a variety of inflammatory adipokines increase arterial stiffening and atherosclerotic risk.
Neuropathically driven dyslipidemia, glucose intolerance, insulin resistance, and reduced metabolic rate accelerate cardiovascular disease risk.
